# Dyslipidemia and risk of renal replacement therapy or death in incident pre-dialysis patients

**DOI:** 10.1038/s41598-018-20907-y

**Published:** 2018-02-15

**Authors:** Pauline W. M. Voskamp, Merel van Diepen, Friedo W. Dekker, Ellen K. Hoogeveen

**Affiliations:** 1 Department of Clinical Epidemiology, Leiden University Medical Center, P.O. Box 9600, 2300RC Leiden, The Netherlands; 2 Department of Nephrology, Jeroen Bosch Hospital, P.O. Box 90153, 5200 ME Den Bosch, The Netherlands

## Abstract

Globally the number of patients on renal replacement therapy (RRT) is rising. Dyslipidemia is a potential modifiable cardiovascular risk factor, but its effect on risk of RRT or death in pre-dialysis patients is unclear. The aim of this study was to assess the association between dyslipidemia and risk of RRT or death among patients with CKD stage 4–5 receiving specialized pre-dialysis care, an often under represented group in clinical trials. Of the 502 incident pre-dialysis patients (>18 y) in the Dutch PREPARE-2 study, lipid levels were available in 284 patients and imputed for the other patients. During follow up 376 (75%) patients started RRT and 47 (9%) patients died. Dyslipidemia was defined as total cholesterol ≥5.00 mmol/L, LDL cholesterol ≥2.50 mmol/L, HDL cholesterol <1.00 mmol/L, HDL/LDL ratio <0.4, or triglycerides (TG) ≥2.25 mmol/L, and was present in 181 patients and absent in 93 patients. After multivariable adjustment Cox regression analyses showed a HR (95% CI) for the combined endpoint for dyslipidemia of 1.12 (0.85–1.47), and for high LDL of 1.20 (0.89–1.61). All other HRs were smaller. In conclusion, we did not find an association between dyslipidemia or the separate lipid levels and RRT or death in CKD patients on specialized pre-dialysis care.

## Introduction

Patients with chronic kidney disease (CKD), defined as an estimated glomerular filtration rate (eGFR) <60 ml/min/1.73 m^2^, have an increased risk of cardiovascular and non-cardiovascular mortality compared to the general population^1^. In the general population dyslipidemia is an important risk factor for cardiovascular morbidity and mortality due to its key role in the development of atherosclerosis^2–4^. Furthermore, dyslipidemia is associated with an increased risk of CKD by accelerating processes that contribute to glomerulosclerosis^5–8^.

CKD patients display a different lipid profile compared to the general population, characterized by e.g. more atherogenic low-density lipoprotein (LDL), low plasma high-density lipoprotein (HDL) cholesterol concentrations with impaired HDL maturation and function, and elevated plasma levels of triglycerides (TG) due to an impaired clearance of VLDL and chylomicrons^9–11^. Hence, dyslipidemia in CKD patients could be a contributing factor to the increased risk on mortality and accelerate kidney function decline.

Observational studies in patients with CKD stage 1–4, thus far, have shown no or a slightly reversed association between dyslipidemia and mortality^12–14^. In contrast, the Study of Heart and Renal Protection (SHARP), an RCT with simvastatin plus ezetimibe in patients with CKD, showed in a sub-analysis in patients (N = 1200) on statin therapy with an eGFR between 15 and 30 ml/min/1.73 m² a significant reduction of 22% of major atherosclerotic events^15,16^.

All in all, the effects of dyslipidemia on start of dialysis and mortality in the later CKD stages remain unclear, while dyslipidemia is a potential modifiable cardiovascular risk factor. Therefore, the aim of the present study is to assess the association between dyslipidemia and risk of renal replacement therapy (RRT) or death among patients with CKD stage 4–5 receiving specialized pre-dialysis care. These data reflect specialized nephrology care and allow us to evaluate the real-world association between dyslipidemia and outcome in pre-dialysis patients, often under-represented or excluded from clinical trials.

## Results

### Patient characteristics

The PREPARE-2 cohort consists of 502 pre-dialysis patients. Of these patients, dyslipidemia could be determined in 274 patients, using the cholesterol measurements (total cholesterol, LDL, HDL, HDL/LDL ratio or TG) from the first 6 months of the study. Of these 274 patients, 181 had dyslipidemia, and 93 did not have dyslipidemia. Baseline characteristics of the patients of the cohort are shown in Table 1. Patients had a median age of 69 (IQR 56–76), 68% were men, 92% were Caucasian, 54% used lipid lowering drugs, and 52% used statins. Patients with dyslipidemia had more often glomerulonephritis as their primary kidney disease, were more frequent a current smoker, had cardiovascular disease as a comorbidity less often, had lower HDL levels, and had higher levels of total cholesterol, LDL, and triglycerides. Of all patients with or without dyslipidemia 50% and 59%, respectively, used a statin at baseline. During the study 91 patients switched statin use from users to non-users or vice versa.

**Table 1:** Baseline characteristics of all 502 pre-dialysis patients of the PREPARE-2 Study and according to the presence of dyslipidemia.

	Total population (n = 502)	Patients with dyslipidemia (n = 181)	Patients without dyslipidemia (n = 93)
Men	341 (68)	129 (71)	55 (59)
Age, years	69 (56–76)	66 (55–75)	70 (59–78)
Ethnicity			
Caucasian	462 (92)	168 (93)	86 (93)
Negroid	29 (6)	11 (6)	2 (2)
Other	11 (2)	2 (1)	5 (5)
Primary Kidney Disease			
Renal vascular disease	154 (31)	60 (33)	30 (32)
Diabetes	72 (14)	20 (11)	14 (15)
Glomerulonephritis	67 (13)	60 (33)	14 (15)
Other	209 (42)	74 (41)	35 (38)
Current smoker, yes	99 (20)	40 (22)	15 (16)
Diabetes Mellitus, yes^a^	130 (26)	45 (25)	25 (27)
Cardiovascular Disease, yes^b^	207 (41)	75 (41)	44 (47)
Hypertension, yes^c^	445 (89)	163 (90)	81 (87)
Systolic blood pressure, mmHg	142 (22)	144 (23)	142 (21)
Diastolic blood pressure, mmHg	78 (12)	79 (11)	77 (11)
Body Mass Index, kg/m²^g^	26 (23–30)	26 (23–30)	25 (23–30)
Subjective Global Assessment^dg^			
Well nourished	336 (67)	120 (66)	58 (62)
Moderately well nourished	43 (9)	13 (7)	9 (10)
Anti-hypertensive drug use	413 (82)	151 (83)	75 (81)
Lipid lowering drug use^eg^	273 (54)	96 (53)	55 (59)
Statin use	263 (52)	91 (50)	55 (59)
Serum creatinine, μmol/L^g^	357 (115)	348 (112)	360 (101)
eGFR, ml/min/1.73 m²^fg^	15 (6)	16 (6)	14 (6)
Total cholesterol, mmol/L^g^	4.45 (1.20)	4.76 (1.19)	3.87 (0.93)
LDL, mmol/L^g^	2.49 (0.93)	2.77 (0.94)	1.87 (0.44)
HDL, mmol/L^g^	1.28 (0.45)	1.22 (0.47)	1.42 (0.38)
Triglycerides, mmol/L^g^	1.52 (1.11–2.20)	1.87 (1.34–2.60)	1.20 (0.98–1.50)
Albumin, g/L^g^	40.7 (4.6)	41.2 (4.5)	41.1 (4.2)
C-Reactive Protein, mg/L^g^	4 (2–9)	4 (3–10)	3 (1–8)
Proteinuria, g/24 h^g^	1.08 (0.37–2.20)	1.09 (0.38–2.23)	1.01 (0.41–1.50)

Values are given as mean ± SD or median and interquartile range (IQR).

^a^Defined as the presence of diabetes mellitus as primary kidney disease or a history of diabetes mellitus. ^b^Defined as presence of coronary artery disease, a history of cardiovascular accident, peripheral vascular disease, or myocardial infarction. ^c^Defined as either a history of hypertension, antihypertensive drug use, a systolic blood pressure ≥140 mmHg or a diastolic blood pressure ≥90 mmHg at baseline. ^d^Defined as well-nourished [subjective global assessment (SGA) 6–7], moderately well-nourished (SGA 3–5), and severely malnourished (SGA 1–2).

^e^Defined as the prescription of statins, fibrates, or cholesterol absorption inhibitors. ^f^eGFR (estimated glomerular filtration rate) is calculated with the CKD EPI (Chronic Kidney Disease Epidemiology Collaboration) formula 2009. ^g^Body Mass Index was known for 492 patients, Subjective Global Assessment for 379, Serum creatinine and eGFR for 438, proteinuria for 247, total cholesterol for 230, LDL cholesterol for 201, HDL cholesterol for 207, triglycerides for 211, albumin for 403, C-reactive protein for 198, and lipid lowering drug use for 398 patients.

### Start of dialysis, renal replacement therapy and death

Of all patients the median follow up time was 66 months (IQR 61–71). During follow up 376 patients (75%) started RRT and 47 patients (9%) died. The crude incidence rate for the combined endpoint RRT or death in patients with or without dyslipidemia was 35/100 py and 31/100 py, respectively (Table 2). For all Cox proportional hazards regression analyses the proportional hazards assumption was fulfilled (plots not shown). The crude and adjusted hazard ratios (HR) for the outcomes for dyslipidemia and for each serum lipid category (outside or within target range) are presented in Table 3. After multivariable adjustment, including statin therapy, the HR for dyslipidemia was 1.12 (0.85 to 1.47). We only found a weak positive association between LDL and RRT or death with a HR of 1.20 (95% CI 0.89 to 1.61) for high LDL compared to a LDL level within target range. We found no association between RRT or death and the lipid levels when analyzed as continuous variables (Table 4).

**Table 2:** Crude incidence rates (95% CIs) of primary outcomes according to the presence of dyslipidemia (n = 502).

	Total (n = 502)	Patients with dyslipidemia (n = 181)	Patients without dyslipidemia (n = 93)
Person years (py)	950.9	337.5	191.7
Start dialysis, n (%)	327 (65)	117 (65)	60 (65)
Incidence rate/100 py	34.4	34.7	31.3
95% CI	23.5 to 47.5	28.7 to 41.5	23.9 to 40.3
Start RRT, n (%)	376 (75)	137 (76)	71 (76)
Incidence rate/100 py	39.5	40.6	37.0
95% CI	28.6 to 54.5	34.1 to 48.0	28.9 to 46.7
Combined RRT or death, n (%)	423 (84)	155 (85)	76 (82)
Incidence rate/100 py	44.5	45.9	39.6
95% CI	32.0 to 59.1	39.0 to 53.8	31.2 to 49.6

CI: confidence interval, n: number, py: person year, RRT: Renal Replacement Therapy.

**Table 3:** Crude and adjusted hazard ratio (95% CI) according to the presence of dyslipidemia, serum lipids or triglycerides category for start of dialysis, RRT and combined endpoint (n = 502).

	Start of dialysis HR (95% CI) (n = 327)	RRT HR (95% CI) (n = 376)	RRT or death HR (95% CI) (n = 423)
*Dyslipidemia*			
No	Ref	Ref	Ref
Yes	1.08 (0.81 to 1.44)	1.11 (0.85 to 1.44)	1.16 (0.90 to 1.50)
Model 1^a^	0.97 (0.71 to 1.32)	1.00 (0.74 to 1.35)	1.07 (0.79 to 1.44)
Model 2^b^	0.95 (0.70 to 1.29)	0.98 (0.72 to 1.34)	1.06 (0.79 to 1.43)
Model 3^c^	1.07 (0.78 to 1.46)	1.06 (0.79 to 1.42)	1.12 (0.85 to 1.47)
*Total cholesterol*			
<5 mmol/L	Ref	Ref	Ref
≥5 mmol/L	0.90 (0.68 to 1.19)	0.95 (0.73 to 1.24)	0.99 (0.76 to 1.30)
Model 1^a^	0.85 (0.59 to 1.21)	0.87 (0.62 to 1.23)	0.92 (0.66 to 1.30)
Model 2^b^	0.84 (0.58 to 1.21)	0.87 (0.61 to 1.24)	0.92 (0.65 to 1.31)
Model 3^c^	0.98 (0.65 to 1.47)	0.98 (0.67 to 1.44)	1.01 (0.70 to 1.46)
*LDL*			
<2.50 mmol/l	Ref	Ref	Ref
≥2.50 mmol/l	1.04 (0.80 to 1.34)	1.10 (0.85 to 1.42)	1.19 (0.93 to 1.50)
Model 1^a^	1.01 (0.76 to 1.32)	1.05 (0.79 to 1.39)	1.13 (0.86 to 1.48)
Model 2^b^	1.00 (0.75 to 1.33)	1.04 (0.78 to 1.40)	1.13 (0.86 to 1.49)
Model 3^c^	1.08 (0.78 to 1.49)	1.11 (0.82 to 1.52)	1.20 (0.89 to 1.61)
*HDL*			
<1.00 mmol/l	Ref	Ref	Ref
≥1.00 mmol/l	1.13 (0.76 to 1.69)	1.06 (0.74 to 1.53)	1.03 (0.73 to 1.46)
Model 1^a^	1.10 (0.72 to 1.69)	1.07 (0.73 to 1.57)	1.02 (0.70 to 1.49)
Model 2^b^	1.10 (0.73 to 1.67)	1.07 (0.74 to 1.55)	1.02 (0.71 to 1.47)
Model 3^c^	1.10 (0.69 to 1.75)	1.08 (0.71 to 1.65)	1.02 (0.69 to 1.51)
*HDL/LDL ratio*			
<0.4	Ref	Ref	Ref
≥0.4	0.96 (0.67 to 1.37)	0.97 (0.74 to 1.27)	0.98 (0.78 to 1.24)
Model 1^a^	1.03 (0.68 to 1.58)	1.01 (0.68 to 1.48)	0.99 (0.69 to 1.44)
Model 2^b^	1.04 (0.69 to 1.56)	1.00 (0.69 to 1.46)	0.99 (0.69 to 1.43)
Model 3^c^	1.00 (0.63 to 1.59)	0.98 (0.64 to 1.48)	0.99 (0.66 to 1.48)
*Triglycerides*			
<2.25 mmol/l	Ref	Ref	Ref
≥2.25 mmol/l	1.00 (0.75 to 1.32)	0.97 (0.74 to 1.27)	0.98 (0.78 to 1.24)
Model 1^a^	0.85 (0.63 to 1.15)	0.84 (0.64 to 1.09)	0.87 (0.67 to 1.13)
Model 2^b^	0.87 (0.64 to 1.17)	0.85 (0.64 to 1.13)	0.88 (0.67 to 1.15)
Model 3^c^	0.91 (0.64 to 1.31)	0.90 (0.65 to 1.23)	0.91 (0.68 to 1.22)

^a^Model 1 was adjusted for; age, sex, ethnicity, current smoker, body mass index, diabetes mellitus, hypertension, proteinuria and primary kidney disease.

^b^Model 2 was adjusted for: model 1 + CRP, Albumin, Subjective Global Assessment.

^c^Model 3 was adjusted for: model 2 + Lipid-lowering medication use.

CI: confidence interval, n: number, RRT: Renal Replacement Therapy.

**Table 4:** Crude and adjusted hazard ratio (95% CI) according to continuous levels of serum lipids or triglycerides for start of dialysis, RRT and combined endpoint (n = 502).

	Start of dialysis HR (95% CI) (n = 327)	RRT HR (95% CI) (n = 376)	RRT or death HR (95% CI) (n = 423)
*Total cholesterol (per 1 mmol/L increment)*	1.00 (0.90 to 1.11)	1.02 (0.92 to 1.12)	1.04 (0.95 to 1.14)
Model 1^a^	0.96 (0.83 to 1.10)	0.97 (0.85 to 1.10)	0.99 (0.88 to 1.12)
Model 2^b^	0.95 (0.83 to 1.10)	0.96 (0.84 to 1.11)	0.99 (0.87 to 1.13)
Model 3^c^	1.01 (0.87 to 1.18)	1.01 (0.88 to 1.17)	1.03 (0.90 to 1.17)
*LDL (per 1 mmol/L increment)*	0.97 (0.83 to 1.13)	1.01 (0.88 to 1.17)	1.05 (0.92 to 1.20)
Model 1^a^	0.95 (0.80 to 1.12)	0.99 (0.84 to 1.16)	1.02 (0.87 to 1.20)
Model 2^b^	0.94 (0.79 to 1.12)	0.98 (0.83 to 1.17)	1.02 (0.86 to 1.20)
Model 3^c^	1.00 (0.83 to 1.21)	1.03 (0.86 to 1.24)	1.06 (0.89 to 1.26)
*HDL (per 1 mmol/L increment)*	0.98 (0.68 to 1.40)	1.02 (0.74 to 1.39)	1.05 (0.79 to 1.41)
Model 1^a^	1.03 (0.72 to 1.48)	1.06 (0.76 to 1.47)	1.09 (0.80 to 1.47)
Model 2^b^	1.03 (0.71 to 1.49)	1.05 (0.75 to 1.48)	1.08 (0.79 to 1.49)
Model 3^c^	1.07 (0.72 to 1.60)	1.09 (0.76 to 1.56)	1.13 (0.81 to 1.57)
*HDL/LDL ratio (per 1 point increment)*	1.01 (0.67 to 1.51)	0.99 (0.69 to 1.41)	0.96 (0.70 to 1.32)
Model 1^a^	1.07 (0.76 to 1.51)	1.04 (0.74 to 1.45)	1.02 (0.74 to 1.38)
Model 2^b^	1.06 (0.74 to 1.52)	1.03 (0.73 to 1.44)	1.01 (0.73 to 1.39)
Model 3^c^	1.01 (0.62 to 1.67)	0.99 (0.62 to 1.58)	0.98 (0.61 to 1.55)
*Tri-glycerides (per 1 mmol/L increment)*	1.02 (0.92 to 1.14)	1.01 (0.91 to 1.11)	1.01 (0.92 to 1.11)
Model 1^a^	0.96 (0.85 to 1.07)	0.94 (0.85 to 1.04)	0.95 (0.86 to 1.06)
Model 2^b^	0.96 (0.86 to 1.06)	0.94 (0.85 to 1.04)	0.96 (0.87 to 1.06)
Model 3^c^	0.98 (0.87 to 1.10)	0.96 (0.86 to 1.07)	0.97 (0.87 to 1.08)

^a^Model 1 was adjusted for; age, sex, ethnicity, current smoker, body mass index, diabetes mellitus, hypertension, proteinuria and primary kidney disease.

^b^Model 2 was adjusted for: model 1 + CRP, Albumin, Subjective Global Assessment.

^c^Model 3 was adjusted for: model 2 + Lipid-lowering medication use.

CI: confidence interval, n:number, RRT: Renal Replacement Therapy.

### Sensitivity analyses

Adding kidney function to model 3 changed the HR for start of dialysis for dyslipidemia to 1.16 (0.80 to 1.67), for RRT to 1.18 (0.82 to 1.69), and for RRT or death to 1.21 (0.87 to 1.69). For LDL ( < 2.5 *vs* ≥2.5 mmol/L) the HR changed to 1.12 (0.60–2.08) for start of dialysis, 1.17 (0.66 to 2.05) for RRT and 1.27 (0.77 to 2.11) for RRT or death. The HRs for high TG were 1.25 (95% CI 0.80 to 1.95) for start of dialysis, 1.20 (0.79 to 1.00) for RRT and 1.17 (0.79 to 1.73) for RRT or death. The HR for the continuous HDL/LDL ratio changed to 0.85 (95% CI 0.45 to 1.58) for start of dialysis, 0.83 (0.49–1.40) for RRT and 0.81 (0.49 to 1.33) for RRT or death. The HRs for total cholesterol and HDL cholesterol, both categorical and continuous, remained unchanged. When stratifying for statin use at baseline, the HRs for dyslipidemia were higher in non-statin users as compared with statin users. the HRs for the separate lipid categories were higher in statin users compared to non-statin users, except for the category exposure HDL and TG and the continuous exposure HDL/LDL ratio. When stratifying for baseline eGFR (≤15 *vs* >15 ml/min/1.73 m²) the HR for the combined endpoint RRT or death was 1.02 (0.68 to 1.53) *vs* 1.36 (0.73 to 2.52) for the presence of dyslipidemia, 0.99 (0.63 to 1.56) *vs* 1.23 (0.72 to 2.11) for total cholesterol, 1.09 (0.75 to 1.59) *vs* 1.46 (0.82 to 2.60) for LDL, 1.10 (0.72 to 1.69) *vs* 0.92 (0.54 to 1.58) for HDL, 0.90 (0.57 to 1.43) *vs* 0.90 (0.53 to 1.56) for triglycerides, and 0.91 (0.55 to 1.51) *vs* 0.92 (0.50 to 1.72) for the HDL/LDL ratio. Restricting the analyses to persistent users and non-users of lipid-lowering medication, the HR for the combined endpoint RRT or death for dyslipidemia decreased to 1.05 (0.77 to 1.43). The HR for the categorical lipid variables increased to 1.33 (0.91 to 1.92) for LDL, decreased to 0.76 (0.51 to 1.14) for the HDL/LDL ratio, and to 0.68 (0.44–1.04) for triglycerides. The HRs for the combined endpoint for total cholesterol and HDL did not change when analyzed as a categorical variable. The HRs for the combined endpoint when analyzing lipids as continuous variables did not change. When we confined all analyses to patients with at least one cholesterol measurement during the first 6 months of their study participation the results did not change essentially.

To account for changes of serum lipids over time, we examined the short term (≤12 months) and long term (>12 months) association between serum lipids and outcomes separately. Tables 5 and 6 show the adjusted hazard ratios for the outcomes for dyslipidemia, and for the serum lipids as a categorical and continuous variable for short and long term follow-up. For dyslipidemia, the short term HRs were lower as compared with the long term HRs, 0.97 (0.63 to 1.49) *vs* 1.23 (0.80 to 1.88) for the combined outcome. For the separate lipid categories and levels short term effects were stronger than long term effects, but without any significant differences since all 95% confidence intervals overlapped.

**Table 5:** Adjusted hazard ratio (95% CI) according to the presence of dyslipidemia, serum lipids or triglycerides category for start of dialysis, RRT and combined endpoint after 12 months and after a minimum of 12 months of follow-up.

	Start of dialysis HR^a^ (95% CI)	RRT HR^a^ (95% CI)	RRT or death HR^a^ (95% CI)
*Dyslipidemia*			
No	Ref	Ref	Ref
Yes			
FU ≤ 12 months	0.88 (0.55 to 1.40)	0.93 (0.59 to 1.47)	0.97 (0.63 to 1.49)
FU >12 months	1.21 (0.75 to 1.95)	1.16 (0.75 to 1.80)	1.23 (0.80 to 1.88)
*Total cholesterol*			
<5 mmol/L	Ref	Ref	Ref
≥5 mmol/L			
FU ≤ 12 months	0.88 (0.55 to 1.41)	0.92 (0.59 to 1.41)	0.97 (0.63 to 1.49)
FU >12 months	1.06 (0.59 to 1.90)	1.04 (0.57 to 1.88)	1.04 (0.61 to 1.78)
*LDL*			
<2.50 mmol/l	Ref	Ref	Ref
≥2.50 mmol/l			
FU ≤ 12 months	1.08 (0.63 to 1.84)	1.13 (0.67 to 1.90)	1.19 (0.72 to 1.98)
FU > 12 months	1.07 (0.61 to 1.86)	1.09 (0.64 to 1.85)	1.20 (0.74 to 1.93)
*HDL*			
<1.00 mmol/l	Ref	Ref	Ref
≥1.00 mmol/l			
FU ≤ 12 months	1.11 (0.62 to 1.97)	1.10 (0.63 to 1.91)	1.04 (0.58 to 1.84)
FU > 12 months	1.15 (0.65 to 2.01)	1.12 (0.68 to 1.85)	1.05 (0.66 to 1.68)
*HDL/LDL ratio*			
< 0.4	Ref	Ref	Ref
≥0.4			
FU ≤ 12 months	0.92 (0.51 to 1.67)	0.87 (0.50 to 1.52)	0.93 (0.56 to 1.56)
FU > 12 months	1.05 (0.59 to 1.89)	1.05 (0.62 to 1.77)	1.01 (0.61 to 1.70)
*Triglycerides*			
<2.25 mmol/l	Ref	Ref	Ref
≥2.25 mmol/l			
FU ≤ 12 months	0.69 (0.36 to 1.31)	0.69 (0.39 to 1.21)	0.71 (0.40 to 1.24)
FU > 12 months	1.14 (0.75 to 1.73)	1.08 (0.74 to 1.58)	1.09 (0.76 to 1.55)

^a^All analyses were adjusted for: age, sex, ethnicity, current smoker, body mass index, diabetes mellitus, hypertension, proteinuria and primary kidney disease, CRP, Albumin, Subjective Global Assessment, Lipid-lowering medication use.

CI: confidence interval, RRT: Renal Replacement Therapy, FU: follow-up.

**Table 6:** Adjusted hazard ratio (95% CI) according to continuous levels of serum lipids or triglycerides for start of dialysis, RRT and combined endpoint after 12 months and after a minimum of 12 months of follow-up.

	Start of dialysis HR^a^ (95% CI)	RRT HR^a^ (95% CI)	RRT or death HR^a^ (95% CI)
*Total cholesterol (per 1 mmol/L increment)*			
FU ≤ 12 months	0.98 (0.81 to 1.18)	0.99 (0.83 to 1.18)	1.01 (0.85 to 1.20)
FU > 12 months	1.03 (0.80 to 1.31)	1.02 (0.81 to 1.28)	1.04 (0.85 to 1.27)
*LDL (per 1 mmol/L increment)*			
FU ≤ 12 months	1.02 (0.78 to 1.34)	1.07 (0.82 to 1.40)	1.08 (0.84 to 1.38)
FU > 12 months	0.95 (0.72 to 1.26)	0.98 (0.76 to 1.27)	1.04 (0.81 to 1.32)
*HDL (per 1 mmol/L increment)*			
FU ≤ 12 months	1.15 (0.69 to 1.93)	1.13 (0.69 to 1.84)	1.20 (0.76 to 1.90)
FU > 12 months	0.99 (0.57 to 1.70)	1.05 (0.66 to 1.66)	1.05 (0.69 to 1.60)
*HDL/LDL ratio (per 1 point increment)*			
FU ≤ 12 months	1.08 (0.53 to 2.18)	1.03 (0.50 to 2.12)	1.04 (0.52 to 2.09)
FU > 12 months	0.96 (0.51 to 1.82)	0.95 (0.57 to 1.58)	0.91 (0.55 to 1.49)
*Tri-glycerides (per 1 mmol/L increment)*			
FU ≤ 12 months	0.88 (0.73 to 1.05)	0.87 (0.73 to 1.04)	0.88 (0.74 to 1.05)
FU > 12 months	1.08 (0.92 to 1.26)	1.05 (0.91 to 1.21)	1.06 (0.92 to 1.22)

^a^All analyses were adjusted for: age, sex, ethnicity, current smoker, body mass index, diabetes mellitus, hypertension, proteinuria and primary kidney disease, CRP, Albumin, Subjective Global Assessment, Lipid-lowering medication use.

CI: confidence interval, RRT: Renal Replacement Therapy, FU: follow-up.

## Discussion

We found no association between dyslipidemia and start of dialysis, RRT or death in incident pre-dialysis patients.

Our finding is in line with several other studies in CKD patients^7,13,17^. The CRIC study (mean age 58 y, mean eGFR 45 ml/min/1.73 m², 42% non-Hispanic black) showed no association between lipoprotein levels and risk of end-stage renal disease (ESRD) after multivariable adjustment^7,17^. Chawla *et al*. found no association between lipid levels and 10-year mortality in 840 CKD stage 3–4 non diabetic patients (mean age 52 y, mean GFR 33 ml/min/1.73 m², 85% white patients)^13^.

Kovesdy *et al*. found in 986 patients (mean age 67 y, mean eGFR 37 ml/min/1.73 m², 40% statin usage) after multivariable adjustment, including controlling for case-mix and malnutrition-inflammation factors, no association between total cholesterol, LDL and TG, and mortality^12^. In our study additional adjustment for malnutrition-inflammation factors did not essentially change the results, which can be explained by the characteristics of our population. Almost all patients of our cohort were well nourished (SGA of 6 or 7), only less than 10% had a moderate nourishment status. Unfortunately, in 25% of all patients the SGA measurement was not performed. Therefore, we cannot exclude that some pre-dialysis patients were severely malnourished. On the other hand, only 2% of all patients in our cohort had a very low BMI <18.5 kg/m^2^. To adjust for residual confounding due to under nutrition we added serum albumin as a nutritional marker to our analyses, which essentially did not change the results.

Our results are in concordance with a recent guideline, stating that CKD patients ≥50 y should be treated with a statin, independent of lipid or triglyceride levels, without aiming at a target level^18,19^. This shows a paradigm shift moving away from LDL based therapy, towards treatment based on atherosclerotic cardiovascular risks. This shift is caused by the lack of evidence that changing lipid levels affects cardiovascular risk in CKD patients^20–22^.

The guideline advice to start statin therapy independent of lipid levels is based on results of subanalyses of large trials^18,19^. For example the SHARP trial, including 9270 patients with a mean eGFR of 27 ml/min/1.73 m² (mean age 62 y, 63% men, and 23% diabetics), showed that patients treated with statins compared to no statins had a 17% lower risk of cardiovascular outcome^15,16^. More advanced CKD may attenuate statin efficacy, as evidenced by negative statin trials in dialysis patients, such as the 4D study and AURORA. A recent meta-analysis by the CCT Collaboration^15,23–25^, including 28 trials - totaling 183,419 patients, studied the effect of statin therapy on major cardiovascular events and death per eGFR category (eGFR ≥60, 45 to <60, 30 to <45, <30 ml/min/1.73 m², including dialysis patients). They found a progressive smaller beneficial effect from statin therapy on major vascular events with decreasing eGFR. In patients with an eGFR ≥60 ml/min/1.73 m² compared to dialysis patients, the beneficial effect of statins on risk of major vascular events was 0.74 (95% CI 0.70–0.79) and 0.89 (95% CI 0.70–1.14), respectively^25^.

Several hypotheses have been suggested to explain the lack of association between dyslipidemia and cardiovascular morbidity and mortality in patients with impaired kidney function. First, uremia may transform HDL into a promotor of inflammation and atherogenesis^26–29^. In addition, Bauer *et al*. found that HDL functionality (HDL cholesterol efflux capacity) is not associated with cardiovascular events in CKD patients (mean eGFR 46 ml/min/1.73 m²)^30^. This is in line with our finding that high HDL level was weakly associated with an increased risk of adverse outcome. If uremia induced, one would expect a dose-response relation, and as a result a decreasing beneficial effect of high levels of HDL in the later CKD stages. After stratification for baseline eGFR (≤15 or >15 ml/min/1.73 m²) we found a HR for the combined endpoint of 1.10 (95% CI 0.72 to 1.69) and 0.92 (95% CI 0.54 to 1.58) for high versus low HDL^30^. Second, although we adjusted for malnutrition-inflammation factors, we cannot exclude residual confounding by coexistent wasting and/or inflammation that led to both lower lipid levels and an increased risk of adverse outcome. Finally, severe atherosclerosis, as present in most pre-dialysis patients, is multifactorial, and might be too advanced to achieve disease regression by lower lipid levels. In addition, non-traditional cardiovascular risk factors such as a disturbed calcium-phosphate balance may play a more important role than dyslipidemia in the progression of atherosclerosis^31–33^.

Main strength of this study is the specific selection of pre-dialysis patients who were treated according to the current CKD guidelines by nephrologists. Pre-dialysis patients form a special group in chronic kidney disease care and cannot be compared to patients in the early stages of CKD. Since no exclusion criteria were used for the PREPARE cohort a wide range of incident pre-dialysis patients were included and all patient information was used to perform the analyses, our results can be generalized to the clinical practice of pre-dialysis care.

This study has limitations. The main limitation of this study are the missing data. Even though we used multiple imputation to deal with this in the best possible way, it is possible that the amount of predictors was insufficient to complete the data. However, in a sensitivity analysis where multiple imputation was restricted to patients with at least one lipid measurement during the first 6 months of study participation, results did not change materially. Second, information regarding the fasting state of patients was not available, resulting in a lack of distinction between patients with fasting or post-prandial elevated TG levels. Finally we used two different methods to measure LDL. Under normal circumstances the Friedewald equation correlates very well with the direct measurement of LDL^34^. However, we cannot exclude the occurrence of chylomicrons or a combination with high plasma TG, both causes of under- and overestimation of LDL levels.

In conclusion, we found no clear association between dyslipidemia and start of dialysis, RRT or death.

## Methods

### Study design and population

The PRE-dialysis Patient Record-2 (PREPARE-2) study is a prospective cohort study of incident pre-dialysis care patients (≥18 y) who had an estimated glomerular filtration rate (eGFR) of less than 20–30 ml/min/1.73 m² and progressive renal function loss. Patients with a failing kidney transplant, who were transplanted at least one year ago, were also eligible for inclusion. The study has been described in detail elsewhere^35^. In brief, patients were recruited in one of 25 nephrology specialized pre-dialysis outpatient clinics in the Netherlands between July 2004 and June 2011. All patients were treated by their nephrologist in accordance with the treatment guidelines of the Dutch Federation of Nephrology, guidelines partly based on the K/DOQI and EBPG guidelines^36–39^. Patients were followed from the start of pre-dialysis care until start of dialysis, kidney transplantation, death or censoring. Censoring was defined as: refusal for further participation, recovery of kidney function, moving to an outpatient clinic not participating in the PREPARE-2 study, loss to follow up or October 2016 (end of follow up), whichever came first. This study was approved by the medical ethics committee or institutional review boards (as appropriate) of all participating centers. Written informed consent was obtained from all patients. All methods were performed in accordance with the relevant guidelines and regulations.

### Demographic and clinical data

Data on demography, primary kidney disease, comorbidities, medication use, and laboratory values were collected during routine visits to pre-dialysis outpatient clinics. These visits took place at the start of specialized pre-dialysis care, at the moment of reaching one of the study endpoints as described previously, and every intermediate 6-month interval. Laboratory data were extracted from the electronic hospital information systems or medical records. The closest laboratory measurement performed within 90 days before or after the date of a visit was appointed to that visit. HDL cholesterol and TG levels were directly measured following standard procedure in the participating outpatient clinics. LDL cholesterol was either directly measured or estimated with the Friedewald equation: total cholesterol – HDL cholesterol – TG/2.2^40^. This formula was not applied in patients with serum TG levels >8.0 mmol/L. Information regarding the fasting state of the patients was not available. The eGFR was calculated using the CKD-EPI (Chronic Kidney Disease Epidemiology Collaboration) formula from 2009, taking into account age, sex, race, and serum creatinine^41^. Hypertension was defined as either a history of hypertension, antihypertensive drug use, a systolic blood pressure ≥140 mmHg or a diastolic blood pressure ≥90 mmHg at baseline^42^. Nutritional status was scored with the Subjective Global Assessment (SGA), a tool that uses medical history and physical examination to create a score ranging from ‘1’ indicating severe protein energy wasting, to ‘7’ indicating a normal nutritional status^43^. Primary kidney disease was classified according to the codes of the European Renal Association-European Dialysis and Transplantation Association^44^. We grouped patients into four classes of primary kidney disease: glomerulonephritis, diabetes mellitus, renal vascular disease, and other kidney diseases.

### Exposure and outcomes

Dyslipidemia was defined as total cholesterol ≥5.00 mmol/L, LDL cholesterol ≥2.50 mmol/L, HDL cholesterol <1.00 mmol/L, HDL/LDL ratio < 0.4, or TG ≥ 2.25 mmol/L. Outcomes were start of dialysis, start of RRT and the combined endpoint start of RRT or death. Start of dialysis was defined as starting hemodialysis or peritoneal dialysis during follow up. Start of RRT was defined as start of dialysis or receiving a kidney transplant during follow up.

### Statistical analysis

Baseline characteristics were presented as mean ± standard deviation (SD) for normally distributed continuous variables, skewed continuous variables as median with interquartile range (IQR). Categorical variables were presented as number and percentages. Total cholesterol, LDL cholesterol, HDL cholesterol levels, HDL/LDL ratio, and TG were used as determinants, and categorized based on the target goals recommended by the Dutch and international pre-dialysis guidelines, being <5.00 mmol/L, <2.50 mmol/L, ≥1.00 mmol/L, ≥0.4, and <2.25 mmol/L, respectively^38,45,46^. Baseline characteristics were presented for the total population and according to presence or absence of dyslipidemia. Absolute crude incidence rates of the primary outcomes were calculated for the total population and separately for patients with and without dyslipidemia.

We conducted Cox proportional hazards regression analysis, obtaining hazard ratios (HR) with 95% confidence intervals (95% CI) to estimate the effect of dyslipidemia and the different components of dyslipidemia on the three primary outcomes. Because dyslipidemia shows its detrimental effects after long term exposure, we studied dyslipidemia as a fixed risk factor at baseline. The separate components of dyslipidemia were analyzed as categorical and continuous variables. Analyses were adjusted for age, sex, ethnicity, body mass index, diabetes mellitus, hypertension, primary kidney disease, proteinuria and current smoking (model 1). In addition to model 1 we also adjusted for malnutrition-inflammation factors: serum albumin, serum C-Reactive Protein, the SGA score (model 2), as well as for lipid-lowering medication use (statin use, fibrate use, or cholesterol absorption medication use) (model 3). Follow-up time was defined as time between baseline visit of the patient and the start of dialysis, RRT, death, withdrawal or end of follow-up (October 2016). The proportional hazard assumption was tested using a log minus log plot. To estimate the median follow up time, a reversed Kaplan-Meier was used.

Multiple imputation was used to avoid bias and to maintain power^47,48^. Missing values of total cholesterol, LDL cholesterol, HDL cholesterol and TG at baseline, as well as potential confounders at baseline were imputed (using 10 repetitions). The imputed data were predicted based on the available information of each patient.

We performed multiple sensitivity analyses to test the robustness of our findings. First, we added kidney function at baseline into the multivariable models. Since kidney function could be in the causal pathway between dyslipidemia and the outcomes we did not add this variable in the main model. Second, we stratified for statin use, because statins may have a pleiotropioc, non-lipid lowering effect, independent of the effect on lipid levels. Third, we stratified for baseline eGFR (≤15 *vs* >15 ml/min/1.73 m²) to study effect modification between kidney functon and dyslipidemia with regard to the outcome. Fourth, we restricted our analysis to patients who were persistent users or non-users of lipid-lowering medication during the entire study period (adjusted for model 3), since changes in lipid-lowering therapy during the follow up period might dilute treatment effects. Fifth, we studied short and long term effects from baseline dyslipidemia seperately by restricting our follow up time to 12 months (short term) and by restricting our analyses to patients who were still in the study after 12 months (long term). Finally, we repeated all analyses applying multiple imputation confined to patients with at least one serum total cholesterol, LDL cholesterol, HDL cholesterol or TG measurement during the first 6 months of their study participation. A p-value < 0.05 was considered statistically significant. All analyses were performed using SPSS version 23.0 for Windows.

### Data availability

All data generated or analysed during this study are included in this published article. The datasets used and/or analysed during the current study are available from the corresponding author on reasonable request.
